# Silence, activate, poise and switch! Mechanisms of antigenic variation in *Plasmodium falciparum*

**DOI:** 10.1111/cmi.12115

**Published:** 2013-02-21

**Authors:** Julien Guizetti, Artur Scherf

**Affiliations:** 1 Unité de Biologie des Interactions Hôte-Parasite, Département de Parasitologie et Mycologie, Institut Pasteur25 Rue du Dr. Roux, Cedex 15, F-75724, Paris, France; 2 CNRS URA2581, Institut Pasteur25 Rue du Dr. Roux, Cedex 15, F-75724, Paris, France

## Abstract

Phenotypic variation in genetically identical malaria parasites is an emerging topic. Although antigenic variation is only part of a more global parasite strategy to create adaptation through epigenetically controlled transcriptional variability, it is the central mechanism enabling immune evasion and promoting pathogenesis. The *var* gene family is the best-studied example in a wide range of clonally variant gene families in *Plasmodium falciparum*. It is unique in its strict selection of a single member for activation, a process termed monoallelic expression. The conceptual advances that have emerged from studying *var* genes show striking common epigenetic features with many other clonally variant gene families or even single-copy genes that show a variegated expression in parasite populations. However, major mechanistic questions, such as the existence of a potential expression site and the identity of transcription factors or genetic elements driving singular gene choice, are still unanswered. In this review we discuss the recent findings in the molecular processes essential for clonal variation, namely silencing, activation, poising and switching. Integrating findings about all clonally variant gene families and other mutually exclusive expression systems will hopefully drive mechanistic understanding of antigenic variation.

## Introduction

Immune evasion is critical for pathogens in order to establish long-lasting infection and ensure effective transmission. The sites of host–parasite interaction are constantly exposed to recognition by the host immune system. Protozoan pathogens have developed a wide range of sophisticated immune evasion strategies such as antigenic variation (Deitsch *et al*., 2009). Parasites actively modify the expression of variant surface proteins in order to remain invisible to the adaptive immune system.

**Fig. 1:**
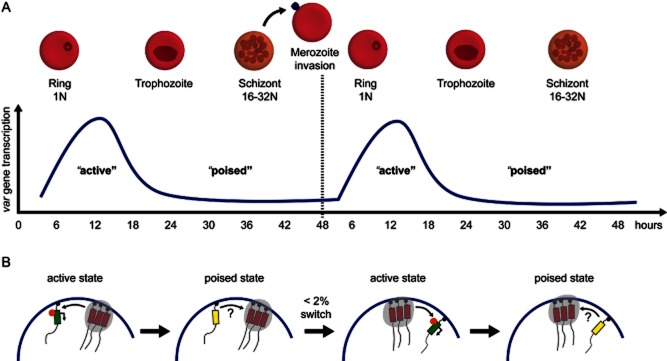
*Var* gene activation and silencing throughout asexual blood stage development of *P. falciparum*. A. The single *var* gene is transcribed at the beginning of the asexual blood stage cycle (ring) soon after merozoites invasion of a red blood cells. Shortly before parasite DNA replication starts, *var* gene transcription ceases but it remains in a poised state (trophozoite and schizont) ready to be activated at the next blood stage cycle. B. All *var* genes, central as well as subtelomeric ones, are tethered to the nuclear periphery (blue) and silent *var* genes (red) cluster in repressive centers (grey) (Lopez-Rubio *et al*., 2009). A single active *var* gene (green) segregates away from repressive centres and forms a perinuclear expression site containing required transcription factors (orange). The transition from the active to the poised state (yellow) is still poorly defined and it is unknown whether positional memory establishes throughout late stages. However, recently a putative methyltransferase PfSet10 has been specifically associated with the poised *var* gene (Volz *et al*., 2012). Variable *var* gene switching rates have been measured *in vitro* (Horrocks *et al*., 2004), but it is unclear at which point in the cell cycle switching occurs.

The protozoan pathogen *Plasmodium falciparum*, which causes human malaria, uses antigenic variation to establish chronic blood stage infections in malaria patients. *P. falciparum* merozoites invade red blood cells and undergo multiple rounds of replication before releasing newly formed merozoites into the blood stream (Fig. 1A). During intracellular development, proteins encoded by distinct clonally variant gene families are transported to the red blood cell surface. Only a small subset of these genes is expressed at a given time. The most extreme form of clonal variation is mutually exclusive expression, also called monoallelic expression. Here, only a single gene of the entire family is transcribed whereas all the others are silenced, as it is the case of the *var* gene family (Scherf *et al*., 2008). The *var* gene family has 60 members and codes for the immunodominant variant adhesion surface molecule ‘*P. falciparum* Erythrocyte Membrane Protein 1’ (PfEMP1). Most *var* genes are in subtelomeric regions, whereas others are arranged in more chromosome central positions. *Var* genes consist of an Exon1, coding polymorphic sequences forming the extracellular domain, an Exon2, coding the semi-conserved intracellular domain, connected by a single highly conserved intron.

Understanding antigenic variation is critical in the fight against human pathogens. In this review we discuss potential mechanisms regulating mutual exclusive expression while focusing on very recent findings. The results from *var* gene expression studies have wide implications for other plasmodial gene families and single-copy genes controlled by similar epigenetic mechanisms (Rovira-Graells *et al*., 2012). Another context where mutual exclusion occurs is the expression of olfactory receptor genes (> 2000 members) in mice (Serizawa *et al*., 2003). Mechanisms investigated there have served as models for *P. falciparum* and *vice versa*.

By default, *var* genes are silenced, which requires the coordination of several distinct epigenetic processes. A single *var* gene is selectively activated for a period of about 10–14 h during the early phase of the 48 h blood stage development (Scherf *et al*., 1998; Schieck *et al*., 2007). Transcription of the activate *var* gene ceases in late blood stage, but despite being silent is epigenetically marked for re-activation during the next erythrocytic cycle, a state called poised (Fig. 1A). Finally, *var* gene inactivation occurs at a low frequency in some cells, leading to the switch to another member of the gene family. Hence, silencing, activation, poising and switching are the basic ingredients that make antigenic variation a successful instrument for immune evasion. We will discuss key factors such as genetic elements, epigenetic chromatin modifications and spatial regulation involved in these four steps. Further, we will highlight outstanding questions in the field and propose future research directions.

## Default silencing

### Epigenetic silencing marks

Though the silent state of *var* genes is maintained over many blood stage generations, it is critical to antigenic variation that each member can be re-activated. In the absence of programmed DNA rearrangements in the activation process (Scherf *et al*., 1998), reversible histone modifications were demonstrated to be decisive to the process of *var* gene regulation (Freitas-Junior *et al*., 2005; Lopez-Rubio *et al*., 2007).

The first functional studies showing an effect on *var* gene silencing involved the inactivation of NAD-dependent histone deacetylase Sir2A (Duraisingh *et al*., 2005). Knocking out *Sir2A* causes de-repression of a subset of *var* genes, especially of upstream promoter sequence (ups) A and B subtypes, as well as some members of the *rifins*, another clonally variant gene family. Inactivation of a second member of the *Sir2* family, called Sir2B, showed some complementary de-repression effect on other, mostly upsC, *var* gene members (Tonkin *et al*., 2009), indicating that both plasmodial *Sir2* genes act on chromatin of clonally variant gene families. Histone deacetylation by Sir2 presumably enables establishment of the silencing heterochromatin mark Histone 3 lysine 9 trimethylation (H3K9me3), which is enriched in promoter regions of repressed *var* genes (Chookajorn *et al*., 2007), including exon1 (Lopez-Rubio *et al*., 2007). Detailed genome-wide Chromatin ImmunoPrecipitation (ChIP) analysis of H3K9me3 distribution in *P. falciparum* has demonstrated an association of the H3K9me3 chromatin mark with multiple gene families including the silent *var* genes and other genes families known to be involved in immune evasion, such as *stevor* and *rifins* (Lopez-Rubio *et al*., 2009). Recently, silencing of the olfactory receptor gene family has also been associated with the heterochromatin mark H3K9me3, suggesting that epigenetic regulation could be a common feature in monoallelic expression (Magklara *et al*., 2011).

H3K9me3 histone modification promotes heterochromatin formation by specific recruitment of Heterochromatin Protein 1 (PfHP1) to silent but not active *var* genes (Perez-Toledo *et al*., 2009). Genome-wide ChIP analysis of PfHP1 showed a strict association with H3K9me3 linking it to the formation of heterochromatin (Flueck *et al*., 2009). To date there is no functional data on how the repressive mark is established but PfKMT1, a member of the SET domain containing family, is a potential lysine methyl transferases candidate (Cui *et al*., 2008).

### Epigenetic silencing in multiple clonally variant gene families

**Fig. 2:**
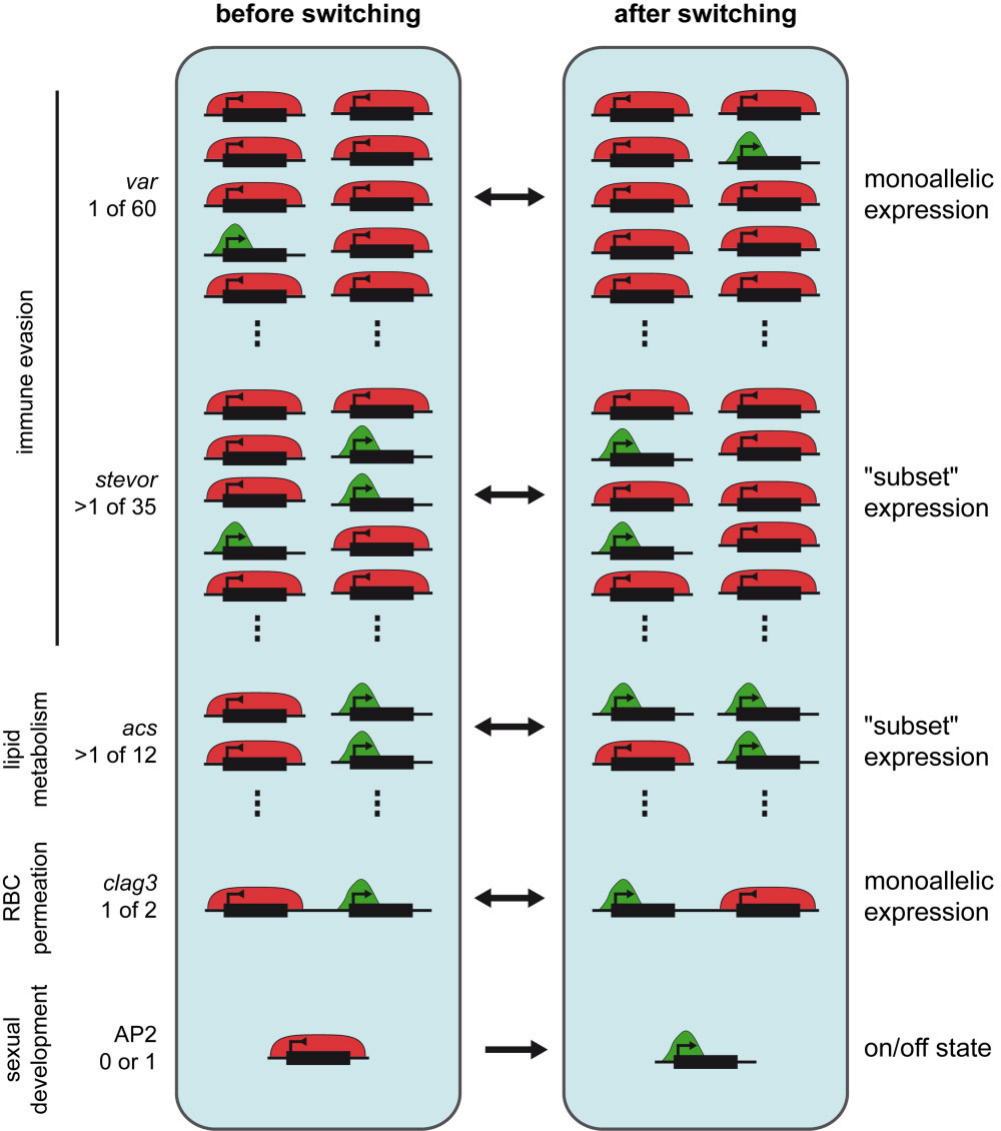
Epigenetic switching in different clonally variant gene families of *P. falciparum*. Several gene families can be differentially expressed in genetically identical parasites (Rovira-Graells *et al*., 2012). The number of active genes (green) varies but silent genes are widely associated with epigenetic silencing mark H3K9me3 (red) (Lopez-Rubio *et al*., 2009). Combinatorial switching of the different gene families can result in a tremendous range of phenotypic variation within genetically identical populations. Gene families involved in immune evasion have different numbers, switching rates and strictness in gene counting. While *var* genes undergo strict monoallelic expression, multiple *stevor* genes can be transcribed in the same cell (Kaviratne *et al*., 2002). Gene families not involved in immune evasion can also undergo clonal variation. Here three examples with different ‘switching modes’ are shown. From the 12 members of the *acs* gene family, encoding for acyl-CoA synthetases 4 members have been shown to be clonally variant in blood stages (Rovira-Graells *et al*., 2012). The *clag3* family, containing two members important for red blood cell permeation pathways, is the only other gene family besides *var* for which mutually exclusive expression has been shown (Cortes *et al*., 2007). A single member of the putative AP2 transcription family (PF3D7_1222600) is associated with H3K9me3. It is thought to be involved in inducing gametocytogenesis (Kafsack *et al*., MPM meeting 2012 Woods Hole). In this simple form epigenetically regulated activation at low frequency could constitute a developmental switch in the life cycle.

Importantly, the large number of gene families showing containing the H3K9me3 mark indicates that clonally variant gene expression is much more widely used by *P. falciparum* than was anticipated (Lopez-Rubio *et al*., 2009; Salcedo-Amaya *et al*., 2010). Unexpectedly, this included also a member of the putative transcriptional regulator gene family AP2 (Apetala 2). Clonally variant transcription in genes involved in processes other than immune evasion has been shown for a receptor protein functioning in erythrocyte invasion (Jiang *et al*., 2010). Further, a detailed genome-wide transcript analysis of freshly cloned parasites revealed the potential for tremendous transcriptional plasticity in genetically identical parasite lines (Rovira-Graells *et al*., 2012). Transcriptional differences could all be linked to genes that show the epigenetic silencing mark H3K9me3 (Fig. 2). Thus, in addition to immune evasion, the epigenetically determined variegated transcription may provide the parasite with another level of adaptation to the variable environments that may occur during infection (bet-hedging strategy). It will be interesting to see whether deciphering *var* gene regulation can help uncover a more generalized transcriptional variation mechanism in other clonally variant gene families and *vice versa*.

### Spatial organization is critical to var gene silencing

Nuclear architecture plays a significant role in the biology of virulence genes in *P. falciparum* (Scherf *et al*., 2008). Telomeres, and consequently subtelomeric *var* genes, cluster at the nuclear periphery by an unknown mechanism. Interestingly, chromosome central *var* genes are also tethered to the nuclear periphery (Ralph *et al*., 2005; Lopez-Rubio *et al*., 2009). A repeat region within the intron of central *var* genes was recently identified as being able to recruit episomes to the nuclear periphery (Zhang *et al*., 2011a). This *var* intron region interacts with a complex of proteins including actin and a member of the AP2 DNA-binding protein family. Pharmacologically induced F-actin formation impairs perinuclear localization of intron-carrying episomes and *var* genes and causes partial de-repression. Hence, tethering to the nuclear periphery seems to be an intrinsic requirement for the default-silencing pathway of *var* genes. Antibodies that label heterochromatin associated molecular markers such as H3K9me3 and PfHP1 are enriched in perinuclear clusters and silent olfactory receptor genes in mice regroup into heterochromatic foci (Clowney *et al*., 2012), supporting the concept of Perinuclear Repressive Centers (PERCs) (Lopez-Rubio *et al*., 2009) (Fig. 1B).

### Var intron as silencing element

Functional gene expression assays using reporter genes highlighted the central role of two *var* genetic elements in expression. The 5′ upstream promoter region and the intron control silencing and activation independently of antigen production (Dzikowski *et al*., 2006; Voss *et al*., 2006). The intron silences expression of transgenes under the control of an episomal *var* promoter, but not other promoters (Deitsch *et al*., 2001; Frank *et al*., 2006). Loss of one-to-one pairing of *var* promoter and intron by episomal recombination in the parasite causes activation of the respective promoter (Deitsch *et al*., 2001; Frank *et al*., 2006). Silencing by strict intron-promoter pairing works downstream as well as upstream of the *var* promoter. Integrating an unpaired promoter into a silent *var* gene cluster leads to its activation, suggesting that the *var* intron plays a direct role, upstream of chromatin spreading, in regulation of *var* gene silencing (Swamy *et al*., 2011). The intron has bidirectional promoter activity transcribing non-coding RNA (Calderwood *et al*., 2003), which may be controlled by the putative Apetala 2 (AP2) domain transcription factor that binds to the central intron region (Zhang *et al*., 2011a). The intron promoter activity is intriguing but the role of this non-coding RNA in *var* gene expression remains unknown.

## Activation

### Epigenetic activation marks

In order to activate a *var* gene, repressive histone marks need to be removed. In the promoter region of the active *var* gene, H3K9me3 is replaced by Histone 3 lysine 4 bi- and tri-methylation (H3K4me2/3) and Histone 3 lysine 9 acetylation (H3K9ac), which renders it permissive for transcription (Lopez-Rubio *et al*., 2007). Consistently, the 5′ ups is no longer associated with PfHP1 when active (Perez-Toledo *et al*., 2009). Histone 4 lysine acetylation is another modification involved in transcriptional activation and potentially regulated by the histone acetyltransferase PfMYST, which is enriched at the active *var* promoter (Miao *et al*., 2010). However, it can be assumed that PfMYST association is not *var* gene specific since overexpression also induces cell cycle defects.

Incorporation of the histone variant H2A.Z around the transcription start site has been associated with increased transcriptional activity (Bartfai *et al*., 2010). In contrast to other developmentally regulated genes, H2A.Z is incorporated in active *var* genes only during ring stages, while it is depleted in later stages (Petter *et al*., 2011). Periodic H2A.Z removal could allow the activation of another *var* gene in the next cycle.

### Var gene promoter region

An episomal *var* promoter expressing a selection marker under drug pressure causes silencing of endogenous *var* genes, demonstrating its infiltration into the mutual exclusive counting mechanism (Voss *et al*., 2006). A similar study, where the construct was integrated into the genome, concluded that the *var* promoter can drive monoallelic expression only when paired with the intron (Dzikowski *et al*., 2006). Discrepancies between those findings have been discussed elsewhere (Dzikowski *et al*., 2007). Nevertheless, those studies suggest that regulatory elements within each *var* gene contain all the necessary information for the singular gene choice upon activation. There seems to be a limiting activation factor for which the active *var* gene is competing (Dzikowski *et al*., 2006). Recently, an eight-base pair sequence motif has been identified in the 5′ UTR of most *var* genes, which apparently is critical for mutually exclusive expression (Brancucci *et al*., 2012). Deleting this mutually exclusive element (MEE) from an episomal active *var* promoter revokes silencing of endogenous *var* genes. This suggests that this element could be the binding site for a limited activating factor.

### Var expression site

Mutual exclusive expression of variant surface glycoproteins (VSGs) in *Typanosoma brucei* occurs at the expression site body, a singular spot within the nucleus defined by the presence of the VSG-specific RNA Polymerase I (Navarro and Gull, 2001). The model claims that this site can only accommodate a single active VSG. This observation has brought forward the hypothesis that a unique and defined expression site for *var* gene transcription could exist in *P. falciparum*. *Var* gene transcription is driven by RNA Polymerase II (Kyes *et al*., 2007), and spatial segregation of the active *var* gene locus and its transcripts from perinuclear repressive centers upon activation has been shown by DNA and RNA fluorescence *in situ* hybridization (FISH) (Freitas-Junior *et al*., 2005; Ralph *et al*., 2005; Voss *et al*., 2006; Lopez-Rubio *et al*., 2009) (Fig. 1B).

Further insights come from exceptional cases where more than one *var* gene is active within a single cell. The HB3 *P. falciparum* strain contains two nearly identical copies of the *var2csa* gene. Despite the fact that one copy lies on chromosome 12 and the other on chromosome 1, RNA FISH analysis showed that simultaneous expression of both copies occurs at the same site in the nucleus (Brolin *et al*., 2009). The Scherf laboratory has recently generated a parasite mutant strain expressing multiple endogenous *var* genes simultaneously at the same spatial expression site (Q. Zhang and A. Scherf, unpubl. data). Interestingly, an episomal promoter of the *rifin* gene families also colocalizes with the active *var* gene expression site when activated through drug selection (Howitt *et al*., 2009). Altogether these observations suggest that there might indeed be a committed nuclear site for expression of clonally variant genes. They prove, however, that this site can, under specific conditions, accommodate more than one active promoter. In yeast, nuclear pores take part in enhanced gene expression (Akhtar and Gasser, 2007). It was tested whether nuclear pores define the *var* gene expression site, or the perinuclear clusters highly expressing 18S ribosomal RNA (Mancio-Silva *et al*., 2010), by colocalization studies combining RNA FISH with immunofluorescence. However, the perinuclear expression site of the active *var* gene, or 18S ribosomal RNA, showed no association with nuclear pores (J. Guizetti *et al*., manuscript submitted). An alternative view is that an activation complex is recruited to the *var* gene *in situ*. This initiates the transcriptional activation cascade including its relocation away from the repressive zone. This scenario predicts that any perinuclear site outside the repressive centres may form an expression site. Depending on which model turns out to be accurate, *var* gene activation would occur before segregation from the repressive cluster or segregation would be a precondition for activation. In either case, the recent discovery of nuclear actin associated to *var* gene intron regions opens a new exciting avenue. Nuclear actin could provide a mechanical framework for spatial organization of active and silent genes (Zhang *et al*., 2011a).

### Enhancer element as monoallelic activator

A different hypothesis for a mutual exclusive expression mechanism postulates the existence of a unique *trans-acting* enhancer element. The discovery of the H-element involved in activation of olfactory receptor genes provides a blueprint for how a single-copy genetic element activates only a single gene through genome-wide interaction in *trans* (Serizawa *et al*., 2003; Lomvardas *et al*., 2006). It is, however, debated whether the H-element does, indeed, mediate activation interchromosomally. Development and adaptation of different genome-wide chromosome conformation capture (3C) analysis for *P. falciparum* would allow testing for such a hypothesis (Simonis *et al*., 2006). A recent study from the Newbold laboratory using genome-wide 3C technology could not detect an enhancer element in monoallelic expression of *var* genes (Lemieux *et al*., MPM meeting 2012 Woods Hole).

## Poising

*Var* genes are only transcribed during the early stages of the blood stage cycle and intrinsic switching rates determined from *in vitro* cultured parasites can reach up to 2% per cycle (Roberts *et al*., 1992). Hence, most of the time the same *var* genes will be re-activated after the next round of invasion (Fig. 1A). ChIP analysis has shown that the activating mark H3K4me2, remains associated with the poised *var* promoter in the late blood cell stages, while H3K9me3 is prevented from spreading into the promoter region (Lopez-Rubio *et al*., 2007). For those marks to facilitate reactivation in the next cycle, they must be maintained throughout S-Phase. Indeed, a H3K4 methyltransferase called PfSet10 colocalizes with the previously active *var* gene locus in post-ring stages, whereas repressing marks are excluded from this site, presumably contributing to the poised state (Volz *et al*., 2012) (Fig. 1B). It is intriguing to speculate that poising might involve nuclear positional memory. In yeast there is evidence for transcriptional memory involving repositioning of genes to the nuclear envelope (Brickner, 2009). The relevance of this topic in *P. falciparum* awaits further investigation.

## Switching

### Var gene switching patterns

Switching between the clonally variant genes is central to antigenic variation and must be adapted to the host so the variant gene repertoire is not depleted too fast whilst effective immune evasion is still possible. It is important to keep in mind that the parasite needs to control its own proliferation so the host is not severely harmed by high parasitemia and effective transmission is guaranteed.

To this day, no genes modifying switch rates have been identified and no functional data is available on the molecular mechanism of switching. Nevertheless, descriptive studies demonstrated that transition rates can vary between *var* members (Horrocks *et al*., 2004). Epigenetic poising of *var* genes during the non-transcribed phase can be experimentally erased by promoter titration (Dzikowski and Deitsch, 2008; Fastman *et al*., 2012). Once memory is erased, the re-activation pattern of *var* genes is independent of previous stages. Another recent study on switch pathways used experimental evidence combined with mathematical modelling to conclude that *var* switching is non-random and necessitates a balanced process of parasite-intrinsic switching and immune-mediated selection (Recker *et al*., 2011). A more detailed transcriptional analysis of *var* gene switching patterns using silencing with drug-selectable markers in multiple isolates suggests that a *var* gene switching pattern is conserved throughout different genetic backgrounds (Enderes *et al*., 2011). Further modelling approaches confirmed that highly structured switching patterns could optimize infection length and robustness (Recker *et al*., 2011). Rigorous statistical analysis and short-term transcriptional data can be used to further improve our models of antigenic switching networks (Noble and Recker, 2012). Parasites having a high dynamic range of on- and off- probabilities within their variant gene families can improve their survival in naïve as well as re-infected hosts.

### Switching and external factors

Another critical question is whether the observed intrinsic switch rate is influenced by external factors, i.e. does the parasite react to its environment, or is the switching pattern hard-wired into the parasite's genome? Although no longitudinal data from human patients are available to support this concept directly, the epigenetic machinery controlling *var* gene silencing may be modulated by external factors. For example, Sir2 depends on NAD levels for its activity. Low nutrition states of patients may influence Sir2 activity and hence alter the switch rate of distinct *var* subtypes. Experimental data from *in vitro* cultured parasites using peroxide or starvation stress can lead to a slight up-regulation of central *var* genes (Rosenberg *et al*., 2009). Elevated PfSir2 levels, which correlate with high temperature and lactate levels, are linked to subtle changes in the *var* gene expression pattern in patients (Merrick *et al*., 2012).

The relative paucity of patient data related to this topic makes it difficult to investigate switching rates in the human host. Two recently published *var* expression studies from malaria patients, however, support the idea that host factors contribute to *var* gene transcriptional control. Unlike *in vitro* cultured parasites, *ups A*-type *var* genes were frequently expressed in *P. falciparum*-infected patients (Bachmann *et al*., 2011; Zhang *et al*., 2011b). Subsequent cultivation resulted in random expression of many *var* genes mostly of the ups B and C-type. It will be crucial to investigate whether host factors modify the intrinsic switch rate of *ups A*-type *var* genes.

## Conclusions

Mutually exclusive expression relies on distinct layers of genetic and epigenetic control factors. Genetic elements, such as the promoter and the intron of *var* genes cooperate with chromatin modifying enzymes in a complex interplay. On top of this, spatial regulation creates specific nuclear sub-compartments most likely critical for default silencing and monoallelic expression. Other factors such as non-coding RNA produced in subtelomeric regions adjacent or within *var* genes may contribute as well to antigenic variation (Epp *et al*., 2009; Sierra-Miranda *et al*., 2012; N. Siegel and A. Scherf, unpubl. data).

Properties and composition of the hypothetical *var* gene expression site remain to be explored. The biggest puzzle, however, in understanding mutual exclusive expression, is the absence of a putative activation factor. The concept of a limiting factor driving monoallelic *var* transcription has been postulated on many occasions but remains to be demonstrated. The notion that a unique enhancer element is the activating factor or expression site body would seem plausible, since there is exactly one copy in the nucleus of this haploid parasite. A transcription factor, whose expression is restricted to extremely low levels, may be considered but it seems difficult to really make sure that exactly one transcription factor is present. Considering that under special conditions more than one promoter can be activated we favour a model where potential positive feedback loops within the activating complex could ensure aggregation around the expressed *var* gene. In the absence of candidate genes for a monoallelic activating factor, identification of mutant parasites that have lost *var* gene expression remains the biggest challenge in the field. Traditional biochemical and mass spectrometric analysis of factors interacting with key genetic elements (Zhang *et al*., 2011a; Brancucci *et al*., 2012; Volz *et al*., 2012) have advanced our knowledge, but technical breakthroughs are needed that will allow identification of molecular complexes of the expression site within the cellular context. Forward genetic screens based on transposition mutagenesis (Balu and Adams, 2006) may reveal unsuspected mechanisms in monoallelic expression. Further, small molecule inhibitors against methyltransferases, as recently developed (Malmquist *et al*., 2012), could provide useful tools to study epigenetically controlled expression of clonally variant genes.

It remains enigmatic how one gene is active while others are silenced, when and how a specific gene is poised rather switched, but many exciting research avenues are left to explore in the field of mutually exclusive expression.
